# Elevating larval source management as a key strategy for controlling malaria and other vector-borne diseases in Africa

**DOI:** 10.1186/s13071-024-06621-x

**Published:** 2025-02-07

**Authors:** Fredros Okumu, Sarah J. Moore, Prashanth Selvaraj, Arnon Houri Yafin, Elijah O. Juma, GloriaSalome G. Shirima, Silas Majambere, Andy Hardy, Bart G. J. Knols, Betwel J. Msugupakulya, Marceline Finda, Najat Kahamba, Edward Thomsen, Ayman Ahmed, Sarah Zohdy, Prosper Chaki, Peter DeChant, Kimberly Fornace, Nicodem Govella, Steven Gowelo, Emmanuel Hakizimana, Busiku Hamainza, Jasper N. Ijumba, William Jany, Hmooda Toto Kafy, Emmanuel W. Kaindoa, Lenson Kariuki, Samson Kiware, Eliningaya J. Kweka, Neil F. Lobo, Dulcisária Marrenjo, Damaris Matoke-Muhia, Charles Mbogo, Robert S. McCann, April Monroe, Bryson Alberto Ndenga, Halfan S. Ngowo, Eric Ochomo, Mercy Opiyo, Richard Reithinger, Chadwick Haadezu Sikaala, Allison Tatarsky, David Takudzwa, Fedra Trujillano, Ellie Sherrard-Smith

**Affiliations:** 1 Environmental Health and Ecological Science Department, Ifakara Health Institute, P.O. Box 53, Ifakara, Tanzania; 2 Institute of Biodiversity, Animal Health, and Comparative Medicine, University of Glasgow, Glasgow, G12 8QQ UK; 3 School of Life Science and Bioengineering, The Nelson Mandela African Institution of Science and Technology, (NM-AIST), Tengeru, P.O. Box 447, Arusha, Tanzania; 4 Vector Control Product Testing Unit (VCPTU) Ifakara Health Institute, Environmental Health, and Ecological Sciences, P.O. Box 74, Bagamoyo, Tanzania; 5 Swiss Tropical and Public Health Institute, Kreuzstrasse 2, 4123 Allschwil, Switzerland; 6 University of Basel, Petersplatz 1, 4001 Basel, Switzerland; 7 Institute for Disease Modeling, Bill and Melinda Gates Foundation, Seattle, USA; 8 ZzappMalaria, Jerusalem, Israel; 9 Pan-African Mosquito Control Association (PAMCA), KEMRI Headquarters, Nairobi, Kenya; 10 Valent Biosciences LLC, Oslo, Norway; 11 Department of Geography and Earth Sciences, Aberystwyth University, Penglais Campus, Aberystwyth, UK; 12 K&S Consulting, Kalkestraat 20, 6669 CP Dodewaard, The Netherlands; 13 Department of Vector Biology, Liverpool School of Tropical Medicine, Liverpool, UK; 14 Malaria Elimination Initiative, University of California San Francisco, San Francisco, USA; 15 Institute of Endemic Diseases, University of Khartoum, Khartoum, 11111 Sudan; 16 Division of Parasitic Diseases and Malaria, Entomology Branch, U.S. President’s Malaria Initiative, U.S. Centers for Disease Control and Prevention, Atlanta, GA USA; 17 DeChant Vector Solutions LLC, 1755 9th St, Columbia, OR 97018 USA; 18 Faculty of Infectious and Tropical Diseases and Centre for Climate Change and Planetary Health, London School Hygiene and Tropical Medicine, London, UK; 19 School of Biodiversity, One Health and Veterinary Medicine, University of Glasgow, Glasgow, UK; 20 Saw Swee Hock School of Public Health, National University of Singapore and National University Health System, Singapore, Singapore; 21 Kamuzu University of Health Sciences, Blantyre, Malawi; 22 National Malaria Elimination Centre, P.O. Box 32509, 10101 Lusaka, Zambia; 23 Clarke International, St. Charles, IL USA; 24 Global Fund Program Management Unit, RSSH and Malaria Grant, Federal Ministry of Health, Khartoum, Sudan; 25 Ministry of Health-Vector Borne and Neglected Tropical Diseases, Nairobi, Kenya; 26 Pan-African Mosquito Control Association (PAMCA), Dar es Salaam, Tanzania; 27 Pesticides Bioefficacy Section, Tanzania Plant Health and Pesticides Authority, P.O. Box 3024, Arusha, Tanzania; 28 Department of Medical Parasitology and Entomology, Catholic University of Health and Allied Sciences, P.O. Box 1464, Mwanza, Tanzania; 29 University of Notre Dame, Notre Dame, IN USA; 30 National Malaria Control Program, Ministry of Health, Maputo, Mozambique; 31 Centre for Biotechnology Research and Development, Kenya Medical Research Institute, Nairobi, Kenya; 32 Kenya Medical Research Institute (KEMRI), Nairobi, Kenya; 33 Public Health Unit, KEMRI-Wellcome Trust Research Programme, PO Box 43640‑00100, Nairobi, Kenya; 34 Center for Vaccine Development and Global Health, University of Maryland School of Medicine, Baltimore, USA; 35 U.S. President’s Malaria Initiative, U.S. Agency for International Development, Washington, DC USA; 36 Centre for Global Health Research, Kenya Medical Research Institute, Kisumu, Kenya; 37 Centro de Investigação Em Saúde de Manhiça (CISM), Maputo, Mozambique; 38 University of California San Francisco Malaria Elimination Initiative (UCSF MEI), San Francisco, USA; 39 RTI International, Washington, DC USA; 40 SADC Malaria Elimination Eight Secretariat, Windhoek, Namibia; 41 Private Consultant, Harare, Zimbabwe; 42 School of Geographical & Earth Sciences, University of Glasgow, Glasgow, G12 8QQ UK; 43 Malaria Modelling Group, School of Public Health, Imperial College London, London, UK; 44 Mwanza University, Mwanza, Tanzania; 45 Rwanda Biomedical Centre, Ministry of Health, Kigali, Rwanda

**Keywords:** Source reduction, Integrated vector control, Core malaria strategy, Larviciding, Community action, Public health, Vector control

## Abstract

Larval source management (LSM) has a long history of advocacy and successes but is rarely adopted where funds are limited. The World Health Organization (WHO) guidelines on malaria prevention recommend the use of LSM as a supplementary intervention to the core vector control methods (insecticide-treated nets and indoor residual spraying), arguing that its feasibility in many settings can be limited by larval habitats being numerous, transient, and difficult to find or treat. Another key argument is that there is insufficient high-quality evidence for its effectiveness to support wide-scale implementation. However, the stagnation of progress towards malaria elimination demands that we consider additional options to the current emphasis on insecticidal commodities targeting adult mosquitoes inside homes. This letter is the result of a global, crossdisciplinary collaboration comprising: (a) detailed online expert discussions, (b) a narrative review of countries that have eliminated local malaria transmission, and (c) a mathematical modeling exercise using two different approaches. Together, these efforts culminated in seven key recommendations for elevating larval source management as a strategy for controlling malaria and other mosquito-borne diseases in Africa (Box 1). LSM encompasses the use of larvicide (a commodity) as well as various environmental sanitation measures. Together, these efforts lead to the long-term reduction of mosquito populations, which benefits the entire community by controlling both disease vector and nuisance mosquitoes. In this paper, we argue that the heavy reliance on large-scale cluster-randomized controlled trials (CRTs) to generate evidence on epidemiological endpoints restricts the recommendation of approaches to only those interventions that can be measured by functional units and deliver relatively uniform impact and, therefore, are more likely to receive financial support for conducting these trials. The explicit impacts of LSM may be better captured by using alternative evaluation approaches, especially high-quality operational data and a recognition of locally distinct outcomes and tailored strategies. LSM contributions are also evidenced by the widespread use of LSM strategies in nearly all countries that have successfully achieved malaria elimination. Two modelling approaches demonstrate that a multifaceted strategy, which incorporates LSM as a central intervention alongside other vector control methods, can effectively mitigate key biological threats such as insecticide resistance and outdoor biting, leading to substantial reductions in malaria cases in representative African settings. This argument is extended to show that the available evidence is sufficient to establish the link between LSM approaches and reduced disease transmission of mosquito-borne illnesses. What is needed now is a significant boost in the financial resources and public health administration structures necessary to train, employ and deploy local-level workforces tasked with suppressing mosquito populations in scientifically driven and ecologically sensitive ways. In conclusion, having WHO guidelines that recognize LSM as a key intervention to be delivered in multiple contextualized forms would open the door to increased flexibility for funding and aid countries in implementing the strategies that they deem appropriate. Financially supporting the scale-up of LSM with high-quality operations monitoring for vector control in combination with other core tools can facilitate better health. The global health community should reconsider how evidence and funding are used to support LSM initiatives.

**Graphical Abstract:**

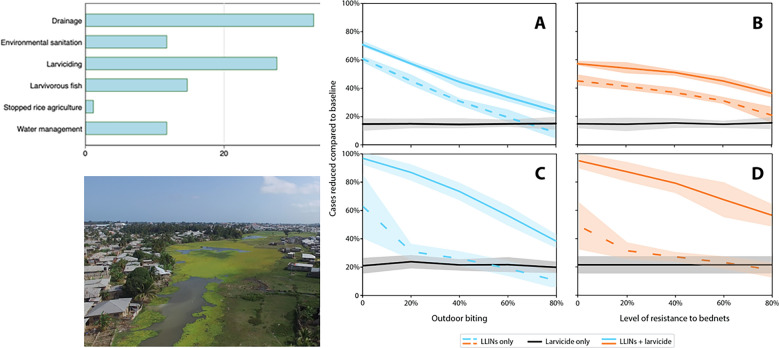

**Supplementary Information:**

The online version contains supplementary material available at 10.1186/s13071-024-06621-x.

## Background

**Figure:**
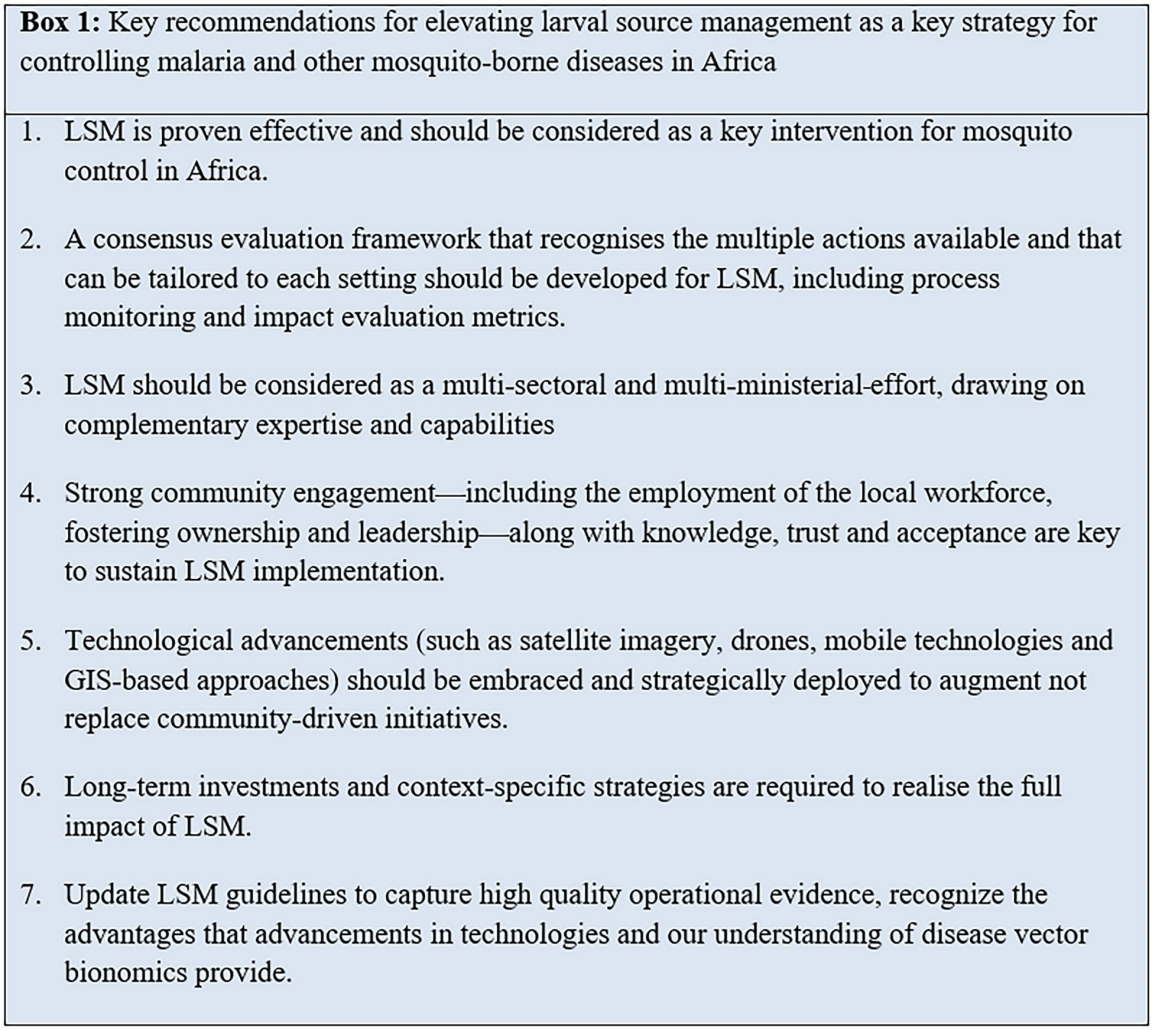

Controlling mosquito vectors is a highly effective strategy to reduce disease burden [1]. Mosquitoes can be targeted either as adults or as larvae in aquatic habitats, with several control strategies available for both life stages. Larval source management (LSM) is an umbrella term that includes larviciding, larval habitat modification (permanent environmental changes or removal), habitat manipulation (continuous effort to make spaces less suitable for mosquitoes) and/or the application of larvivorous fish (or other natural predators for biological control)—all of which aim to reduce the larval habitats available to mosquito vectors, and thus suppress their densities.

The World Health Organization (WHO) develops guidelines for vector control against malaria transmission based on consolidated information that incorporates systematic reviews of the available evidence for intervention effectiveness and pre-existing WHO recommendations. It recommends some interventions for large-scale deployment and others as supplementary [2]. Only those that show proven protective efficacy to “reduce or prevent infection and/or disease in humans at the individual level, community level or both” and are “broadly applicable for populations at risk of disease in most epidemiological and ecological settings” obtain full recommendation for large-scale deployment. So far, this list consists of only insecticide-treated nets (ITNs) and indoor residual spraying (IRS) with insecticidal products and formulations that are WHO-prequalified. WHO has given larviciding a conditional positive recommendation but with low-certainty evidence. This is supported, in part, by a recent Cochrane review on larviciding, which included four eligible studies. Only one of these studies was a cluster-randomized controlled trial (CRT) [3], which are broadly considered the optimal design for generating empirical data on epidemiological impacts. The lack of such studies on LSM efforts is principally due to limited funds, as such trials would likely need to be substantially larger than those conducted for household interventions. Unlike human drug trials or household-level vector control, each LSM context is relatively unique, often applying multiple approaches to reduce larval habitats. Like housing developments, LSM efforts will restructure the local landscape from the vector perspective relative to pre-intervention, perhaps permanently. The best strategies to implement may differ between locations but collectively contribute to reduced vector density and thus reduced disease transmission. As noted by others, CRTs were designed for biomedical interventions and are ill-suited to interventions that alter the environment experienced by the vector in nuanced ways [4]. The current framework from WHO to generate full recommendation will therefore not fit for LSM for this reason, but the opportunity to adopt tailored LSM strategies should not be limited as a consequence. Interestingly, IRS, which was the principal intervention in the first global attempt to eradicate malaria, has long received a strong positive WHO recommendation for large-scale deployment in Africa without any evidence from properly designed large-scale epidemiological trials [5]. This decision was justified based on the documented impact of IRS in multiple historical operational campaigns from the 1950s to the 1980s [6–8] and various national programmatic deployments of IRS.

The WHO guidelines also highlight concerns about feasibility—invoking the principle that LSM is most effective when aquatic habitats are “few, fixed, and findable.” The interpretation of this concept has notably constrained its application, as the typical larval habitats of some African vector species can be numerous, dispersed and difficult to locate, especially during the rainy seasons. Consequently, the application of LSM has been significantly restricted and its use largely limited to urban and arid environments, where the vector habitats are indeed broadly few, fixed and findable. While LSM may not be applicable in some contexts, the current guidelines are too restrictive, and there are notable priority cases that may make LSM more attractive, such as (i) vector control in densely populated semi-urban or urban spaces with lower surface areas of larval habitat that permanently holds water year-round [9], (ii) targeting sites that retain water during drier seasons [10], and (iii) applying efforts to explicit problems, as is being explored in rice cultivation [11]. However, recent advances in technology, including simpler and low-cost mapping technologies, mobile technologies, digital information capture and delivery, and aerial spraying and imaging systems, have increased the feasibility of identifying habitats and implementing an effective LSM programme in a wider range of contexts [12].

After years of decreasing malaria rates in Africa, progress has now stalled, and many countries are reporting rising case numbers [13]. The indoor insecticidal interventions, ITNs and IRS, are faltering due to a number of challenges, including widespread insecticide resistance [14, 15], short lifespan [16], other biological challenges such as mosquitoes biting outdoors or outside sleeping hours [17, 18], high costs of implementation and suboptimal usage in some settings [19]. Although many innovations are underway to bridge these gaps, LSM, a historically proven approach, remains underused. Endemic nations continue relying heavily on commoditized vector control with ITNs and IRS—that can be reported to donors as units delivered, which provide short-term gains but must be regularly replenished at ever increasing costs as the population living in malaria endemic settings continues to increase. Yet the categorization of LSM as a supplementary tool continues to stifle investment in this space and dampen its potential impact. A recent global review of national malaria programmes implementing vector control highlights limited funding to specifically increase numbers and capacity of programmatic staff as a critical roadblock toward shifting countries from a status of “controlling” to “eliminating” malaria [20]. Only 14 of the 35 countries participating in the review were implementing LSM, many at small scale, with inadequate funding, inadequate human resources, logistical challenges and poor infrastructure identified as the critical barriers [20].

The work in this paper was initiated through online discussions and inputs shared through a Google Doc, and joint virtual meetings to reach a consensus on key recommendations. We have included a narrative review of the literature from countries that have previously eliminated malaria, and we used two different transmission models for malaria to theoretically assess the potential of LSM. In this paper, we argue that LSM merits re-evaluation for its potential to reduce malaria and other vector-borne diseases. We highlight the substantial evidence supporting its epidemiological impact, summarize its benefits and weaknesses, and propose key considerations (Box 1) for scaling it up in low-income and rural settings including those with stable year-round disease transmission. Additionally, we identify critical steps to renew investment and recognition for LSM as an essential component of the intervention packages for controlling and eventually eliminating vector-borne diseases, particularly malaria.

## The challenge of evaluating LSM compared to commoditized vector control

The WHO recommends considering supplementary interventions only after large-scale interventions achieve optimal coverage [2]. Full recommendations are restricted to interventions with evidence from systematic reviews of (primarily) cluster-randomized epidemiological trials, limiting the recognition of those with extensive empirical (but non-randomized) studies. This policy has favoured readily commodifiable interventions, especially ITNs, which have been rigorously evaluated with well-designed CRTs, thus discouraging the uptake of traditional evaluation methods predating CRTs. Unlike ITNs, and to a lesser degree IRS, LSM involves diverse implementation strategies that need to be ecologically tailored, making it difficult to define a single consistent, quantifiable metric for process, impact evaluation and extrapolation to other locations—that is, an LSM action may have internal validity for one space but not external validity for other locations [21]. The diversity of local ecologies and differences in environmental sanitation strategies further complicate the measurement of individual- or household-level effects, as LSM primarily offers community benefits rather than impacts that can be measured as personal protection as well as community benefit. In the context of current WHO requirements, these challenges in quantifying the public health impacts of LSM further contribute to the hesitancy in fully endorsing the strategy. This difficulty also reduces donor support due to the variability in efforts or strategies required, and the associated capacity strengthening in entomology that would be needed. These restrictions were overcome for IRS in 2006 when the President’s Malaria Initiative (PMI) orchestrated huge investment into IRS, expanding programmes and training thousands of people with no prior IRS experience [22], so there is precedent for alternative routes. An alternative framework for evidence generation is necessary to demonstrate and compare protective efficacy at both the individual and community levels [4], but one that also facilitates the accelerated adoption of LSM beyond an expectation that a particular style of LSM would be proven successful everywhere—it likely will not. There are parallels between this challenge and that of effective water, sanitation and hygiene (WASH) systems. Researchers working on WASH have also raised concerns that the reliance on CRTs for proving impact from interventions may limit the availability of funding toward implementation or adoption of such complex actions with multi-sectoral benefits [23].

Mosquito control programmes, which tend to differ from externally funded trials, aim at reducing mosquito sources by all relevant cost-effective means, and never just one way. The diversity of LSM approaches has led to studies that explicitly distinguish between commodity-based larviciding and alternative LSM options in our evaluation processes. While evidence for the effectiveness of non-commoditized LSM approaches remains limited [24, 25], a recent Cochrane review of the available data suggests that habitat modification and manipulation interventions for malaria prevention show some indications of benefit in both epidemiological and entomological outcomes [26]. The authors noted that the evidence was mixed, and further studies were needed, but they concluded that these varied approaches may be useful in certain circumstances [26]. Given the sensible approach of programmes to adopt all feasible strategies when integrating source reduction efforts into vector control, establishing a robust evidence base for LSM from high-quality monitoring of operational implementation could encourage greater financial support and achieve scaled LSM applications. Additional field evidence, whether observational or otherwise, could complement the theoretical benefits already demonstrated in modelling exercises [27, 28].

ITNs and IRS are now the dominant forms of vector control in Africa, though use of the latter is decreasing rapidly. Consequently, when CRTs are conducted, LSM will likely always be used alongside other standard-of-care interventions, usually ITNs. When considering this evidence for policy decisions, it is essential to understand such evidence as reflective of only settings where LSM is deployed as a complementary intervention (see [29]). Evidence from such trials has been considered previously in efforts to identify and support the employment of the most cost-effective intervention within the time frame of the trial. The cost per case averted per unit time gives a useful comparative metric but can fail to show the combination of efforts required to reach elimination status. Discovering this, and then strategizing on how to make such action most affordable, is a potential alternative. Moreover, decision-makers should avoid demoting LSM because of an absence of studies that isolate its impact, or the complexity and cost of trial designs required to attribute impact to a particular entity. Indeed, when habitat modification or source reduction options are tested, any short-term trials (e.g. 1 to 3 years, reflecting typical CRT funding periods) will likely underestimate the long-term cost-effectiveness of efforts achieving permanent change [21]. Lastly, LSM is not a solely malaria-centric strategy, and trials focused solely on malaria transmission may overlook parallel or broader community benefits, such as improved sanitation (e.g. removal of tires or rubbish from waterways) and reduced densities of nuisance biting insects and vectors of other diseases.

Most malaria-endemic countries already mention LSM, primarily larviciding, in their strategic plans. The status of LSM within the WHO guidelines may be limiting because it reduces the strategy to a lower priority than other interventions, meaning that there is potential for both countries and donors to consider it a lower priority where funds are limiting. One of the major donors, the Global Fund, can support LSM, as they can support interventions that are recommended in the WHO guidelines for malaria. This includes both interventions with a “strong recommendation for” and those with a “conditional recommendation for”. LSM falls into the “conditional” category—as do several other things that the Global Fund support (e.g. pyrethroid-piperonyl butoxide [PBO] nets). Countries make grant applications for funding and, following a review process that considers what countries wish to prioritize, the position of the funder and outcomes from a Technical Review Panel and Grant Approval Committee, support is provided. Currently, however, budget is rarely offered for LSM at the end of this process. Despite the conditional recommendation, national malaria programmes are already using or advocating for LSM independently. Beyond the core guidelines, LSM is also recommended by WHO and other agencies as a key strategy for controlling the spread of the invasive *Anopheles stephensi* that share the breeding habitat with *Aedes aegypti* in major water containers, which is increasingly spreading in Africa, especially in urban areas [9, 30].

Tanzania is a notable example of how malaria-endemic countries can mobilize local resources to support LSM. The government formed the End Malaria Council during World Malaria Day in 2023 (https://www.afro.who.int/countries/united-republic-of-tanzania/news/tanzania-forms-end-malaria-council-malaria-day-2023), which will be used to mobilize funds to support the implementation of LSM and other interventions requiring additional funding. The government is also allocating funds to support local administrative councils in procuring bio-larvicide, which is locally manufactured by the government-supported plant, Tanzania Biotech Product Ltd. Additionally, the councils are encouraged by the President’s Office, Regional Administration, and Local Government (PO-RALG) to allocate funds to support the implementation of bio-larviciding. The plan by the Tanzania National Malaria Control Program (NMCP) to involve the Vector Control Technical Working Group members in supporting the planning and proper implementation of LSM is crucial for monitoring and maximizing the impact of LSM, while also providing an opportunity to conduct operational research.

There are notable cases where the effectiveness, feasibility and scalability of LSM as a key intervention have been demonstrated. For example, in the Khartoum malaria-free initiative between 1995 and 2004, substantial progress in malaria control was achieved through the weekly application of temephos larvicide and environmental management [31]. This success in an endemic, low-income setting is further supported by substantial evidence from India [see [32], and references therein]. Ultimately, WHO guidelines that are broadly supportive of habitat modification efforts would encourage the use of the various LSM opportunities that exist and help to increase funding.

## The case for expanding the application of LSM in Africa

Interventions recommended for mass deployment (e.g. ITNs and IRS), even when deployed at optimal coverage, leave significant gaps in protection at the individual and community levels [13, 33–36]. We argue that a multifaceted approach that integrates LSM alongside other vector control strategies—as has been done historically (see Supplementary File 1)—can significantly reduce vector populations and offset key biological threats such as insecticide resistance and outdoor biting. Further, environmental modification critically prevents re-establishment of vector populations, thus advancing malaria control and elimination across geographic and socio-economic landscapes.

### Evidence of epidemiological impact

**Fig. 1:**
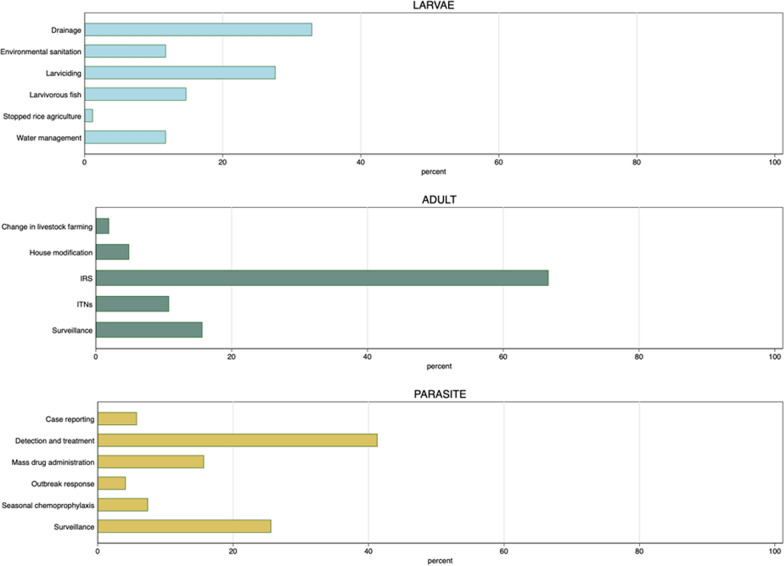
A summary of a narrative review of the countries that have achieved WHO-recognized elimination of malaria transmission locally (see Supplementary File 1). The proportion of countries that have used a noted approach during their path toward elimination is shown and the stage of the parasite noted

The public health impacts of LSM have been regularly reviewed [32, 37–40], and the evidence from both historical and recent LSM applications can be considered by countries to inform deployment of the strategy in context-specific settings while learning in the process. Our narrative review of countries that previously eliminated malaria reveals that nearly all of them used some form of LSM (out of 85 countries reporting on any efforts, 73 note LSM), often involving habitat manipulation or modification (Fig. 1 and Supplementary File 1). Almost every country in Europe, the Middle East, USA and China used drainage and habitat modification. While more challenging in the tropics, these approaches were achieved in several countries. Notably, Cape Verde—which was recently declared malaria-free by WHO (in 2024)—used temephos larvicide, larvivorous fish and environmental management in addition to IRS and case detection [41, 42]. Indeed, LSM, when meticulously and intensively executed at large scale, usually in combination with effective case management and enacted as an integrated mosquito management (IMM) exercise, has proven potent in mitigating, and occasionally contributing to the local elimination of, key vector populations [43].

The most notable campaigns documented to have entirely suppressed malaria vectors occurred across 54,000 km^2^ of the northeast coast of Brazil [44], in a 1000-km stretch of the Nile Valley in Egypt [45], around a Zambian copper mine [46], in the USA, Palestine and Israel, and the Italian elimination of malaria [47]. In both Brazil and Egypt, the impacts were primarily due to vertically directed “military-style” larval control efforts aimed at removing all live specimens in relatively short time frames, with the primary objective being vector extermination rather than disease control. The Brazil example was particularly unique as it not only involved the use of simple paper maps and larvicides (Paris Green) but was also completed at a time of war with likely consequential supply chain interruptions. In Zambia, extensive environmental modification was combined with sustained larviciding, treatment, net use, house screening, and eventually DDT spraying [43, 46, 48, 49]. This reduced malaria incidence by over 95% in approximately 10 years, with an 88% reduction in deaths [49]. Kitron and Spielman [47] report that the “modification or elimination of aquatic habitats for mosquito breeding” proved decisive in the USA, Palestine, Israel and Italy. In Dar es Salaam, habitat manipulation was at least as effective as ITNs, and LSM combined with ITNs was synergistic and more effective than ITNs alone [50]. This is particularly important because CRTs have indicated that even with high coverage, the best intervention on the market—pyrethroid-pyrrole ITNs—was not enough to drive down malaria and sustain control in Benin [51] or Tanzania [52]. In rural Kenya, LSM significantly enhanced the protective effect of ITNs by reducing vector populations [53, 54]. In the Khartoum malaria-free initiative, without IRS or ITNs, malaria was reduced from 24% to less than 1% using different LSM interventions including chemical larviciding, environmental management (mainly intermittent irrigation in small farms surrounding the city) and strong coordination with related sectors and community participation including the engagement of school pupils [55]. Elsewhere, in Nigeria, malaria incidence was reduced by nearly 80% in 1 year following habitat drainage [56]. Recent evidence from rural Northern Côte d’Ivoire showed a 40–50% reduction in malaria incidence with *Bacillus thuringiensis israelensis* (Bti) larviciding, an impact that is comparable to dual-active-ingredient ITNs [29]. This suggests that concerted efforts could provide at least an equivalent impact to that measured for next-generation ITNs in recent CRTs [51, 52, 57, 58].

### Impacts on multiple vectors and nuisance biters

The Global Vector Control Response Framework [59] advocates for targeting multiple mosquito species simultaneously through the use of LSM and other vector control approaches. However, the segregation of institutional efforts focused on controlling specific mosquito-borne diseases, such as malaria or dengue, often prevents the full potential of LSM from being realized or measured. While the malaria burden in Africa is well documented [13], other mosquito-borne diseases, particularly those borne by *Aedes* mosquitoes, remain poorly understood and poorly addressed [60]. Moreover, climate change is expected to exacerbate the prevalence of these *Aedes*-borne diseases, making integrated vector control increasingly important, especially in urban settings [61, 62]. This includes areas impacted by the invasive *An. stephensi*, where LSM can address both malaria vectors and arbovirus vectors [63], particularly given that species often share the same larval habitat [64].

Lessons can be learned from mosquito control practices outside sub-Saharan Africa, where IMM is critical to addressing public health needs including nuisance biting, and also animal- and crop-protection needs. For example, the US Centers for Disease Control and Prevention (CDC) advises mosquito-abatement districts to implement IMM, which includes controlling mosquitoes as larvae, removing breeding habitats, using structural barriers (such as screening windows) and controlling adult mosquito populations through aerial spraying [65]. Similar actions reduce disease transmission and nuisance biters in Australia [66], Europe [67] and Canada [68]. Although many of these programmes are not directly targeted at malaria, they offer valuable lessons in public health administration and logistical deployments that can support African mosquito control programmes. Such integration not only cuts costs but also leverages expertise from different departments to comprehensively address the challenge, thus fulfilling the WHO definition of integrated vector management (IVM): “*a rational decision-making process to optimize the use of available resources for this strategic intervention*” [69].

### Management of insecticide resistance and residual malaria transmission

Since the 1950s, malaria control strategies have predominantly focused on reducing the daily survival rate of adult mosquitoes, which is generally considered the most critical parameter to target in order to effectively curb malaria transmission [70]. However, behavioural adaptations, such as outdoor or early biting [18], and physiological changes such as insecticide resistance [14] can significantly reduce the protective efficacy of ITNs and IRS. Although novel commodities like dual-active-ingredient treated nets attempt to counter these evolving challenges by effectively targeting pyrethroid-resistant adult vectors, they are insufficient and do not completely mitigate evolving insecticide resistance [51, 52]. In contrast, well-implemented LSM can effectively suppress vector populations at their source, regardless of species composition, behavioural variations or insecticide resistance. Resistance management efforts aim to reduce reliance on a single mechanism of action—which can be done given the range of larvicides already developed—and there is evidence that where resistant phenotypes exist, fitness costs may include extended larval development time, offering potential complementarity from LSM [71]. Even without reducing the survival probabilities of adult mosquitoes or directly impacting sporozoite infection prevalence, significant declines in overall vector populations would lead to a reduction in human biting rates and directly impact vectorial capacity [72], which has recently been (re)considered a more useful metric for planning, monitoring and evaluating vector control [73].

**Fig. 2:**
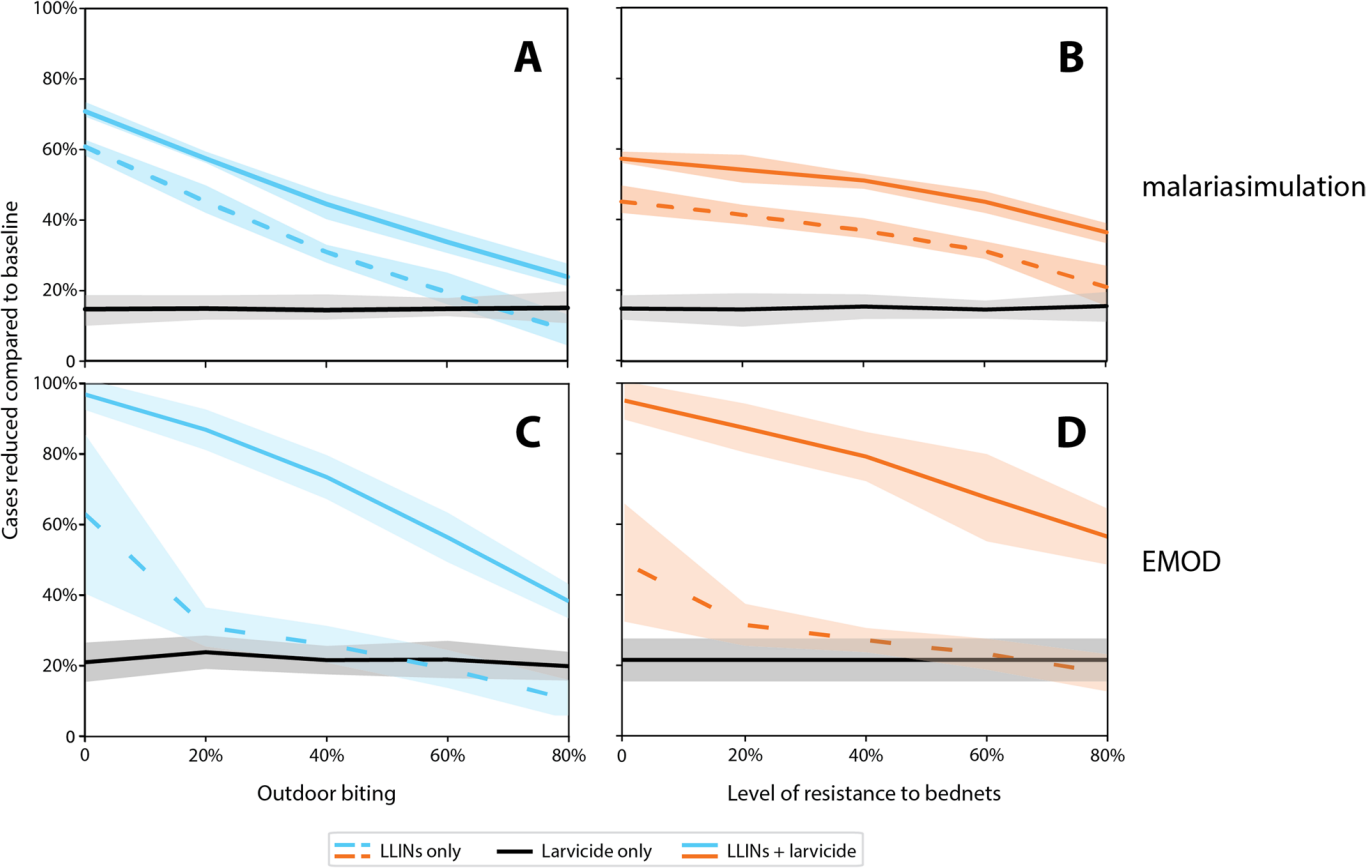
Percentage reduction in clinical cases compared to a baseline scenario with no interventions (**A**, malariasimulation; and **C**, EMOD) for different outdoor biting proportions, and (**B**, malariasimulation; and **D**, EMOD) at different levels of resistance to mosquito insecticide-treated nets (ITNs). Dotted lines indicate a scenario where only bednets are distributed, while solid lines indicate a combination of bednets and larvicide (or LSM, malariasimulation) being distributed. The black solid line shows a scenario with only larvicides with fortnightly application and waning effects (EMOD) or a form of LSM (malariasimulation) that sustains source reductions of 25%. Error envelopes (shaded areas) around the mean (lines) are represented by one standard deviation across 20 stochastic seeds per parameter set (EMOD), and stochasticity across 20 random seeds for a population of 20,000 people (malariasimulation)

To illustrate this, we compared outputs from two mathematical modelling frameworks, malariasimulation [74] (Fig. 2A and B) and EMOD [epidemiological modelling] (EMOD v2.20, 2024) (Fig. 2C and D). In both transmission models, we assumed seasonal malaria in a generic setting and an effective entomological inoculation rate (EIR) of 90 infectious bites per person per year in the absence of interventions. Intervention scenarios were simulated for 3 years, with ITN distributions occurring every 3 years per WHO recommendations [75] and ITN use simulated to reach 70% immediately after the mass campaign before waning over 4 years. The way that interventions are implemented differs between the models. Simulations using EMOD, an agent-based mechanistic model of malaria transmission with vector life cycle [76] and within-host parasite and immune dynamics [77], assume ITN killing starts at a maximum 70%, and loss in efficacy due to insecticide resistance is modelled by varying this value. Irrespective of resistance, loss in killing efficacy over time due to washing and general decay of insecticide is simulated using an exponential decay rate of 2 years. ITN blocking starts at 90%, with an exponential decay rate of 2 years to model the physical integrity of nets for all scenarios [16]. In malariasimulation, the maximum mortality impact due to ITNs reaches 32.7%, with repellence at 64% for mosquitoes attempting to feed while a person is in bed. The mean duration of impact is 3.8 years, waning exponentially, and insecticide resistance reduces both the potency and duration of this impact. These parameters are derived from systematic reviews of experimental hut data and validated by simulating CRTs on ITNs [78–80] and updated by Churcher et al. [81].

For larvicides, the EMOD model (EMOD v2.20, 2024) assumes a biolarvicide (containing Bti and *Bacillus sphaericus* [Bs]) that starts with a killing efficacy of 85%, remaining stable for 5 days and then decreasing with an exponential decay rate of 1 (Msugupakulya et al. Unpublished data). In all simulations, larvicides were distributed at 60% coverage of productive habitats, with repeated sprays every 14 days over 4 months. Larviciding was assumed to be carried out in the second year after ITN distribution to account for reduced usage of nets a year after distribution as well as asynchronous distribution of nets leading to lower overall efficacy in a population. In malariasimulation, LSM is implemented to reduce adult densities by 25% and sustain this suppression regardless of the LSM strategy used (i.e. this could be the impact from application of a larvicide, or drainage or other method of source reduction). No loss of impact is simulated.

In both models, in the scenario with varying outdoor biting (Fig. 2A and C), simulations were run without loss in ITN killing efficacy due to insecticide resistance. In the scenarios with varying resistance intensity, outdoor biting was held at 20% (Fig. 2B and D). The number of clinical cases was calculated across a year starting from 1 year after the start of larviciding (i.e. results represent the overall reduction in clinical cases during the second year of an ITN campaign), and all simulations are compared to a baseline case without any vector control interventions. Model parameters and summary outputs are provided in Supplementary File 2.

While the models have slightly different scales of impact according to their specific functional associations determining malaria transmission, and make explicit assumptions on ITN and LSM impacts that would need to be empirically confirmed [see [80] for ITN impact validations for malariasimulation], they show similar expectations on ITN-only impacts, and that LSM can be agnostic to the level of insecticide resistance in local mosquitoes or residual transmission, including mosquitoes biting outside the effective times of indoor interventions. If LSM is not itself applying an insecticide, this may hold true for all forms of insecticide resistance against different insecticides and classes. Even though LSM does not directly reduce the survival of adult mosquitoes, it intrinsically decreases the population of mosquitoes entering the transmission system, consequently reducing the number of bites per person. Therefore, when used alongside current adult-targeting interventions like ITNs and IRS, LSM can potentially enhance malaria control efforts, even in areas with significant levels of resistance. An overarching challenge now is determining how to measure the impacts of LSM efforts and designing metrics that allow for sharing advice across variable settings. Clearly, LSM can address mosquito populations with mechanisms completely different from those used to control adult mosquitoes. Moreover, LSM offers an opportunity for integrated vector control, enabling vector control campaigns to remain effective regardless of resistance patterns.

## Working with community members to ensure equity, acceptability and overall effectiveness

We recognize that LSM will not be universally appropriate [82]. One metric that could be useful is the ratio of human population density to the surface water area requiring treatment. LSM might prove highly effective in peri-urban areas where there is a high human-to-surface water ratio, but less so in rural settings where this ratio is low. Nonetheless, we emphasize that LSM has the potential to offer equitable protection against malaria in various settings, potentially more regions than previously suggested by WHO recommendations, which mainly focus on urban or arid environments [2]. Its added value is particularly evident in areas where vector species are infective but where conditions make ITN use uncomfortable or unfeasible; where people engage in outdoor activities overnight, in the evenings, and early mornings; where certain demographic subsets are consistently unprotected [83]; or where the costs of current mosquito control commodities are untenable. The equitable protection afforded by LSM for all community members reduces the social hierarchical challenges associated with other interventions, such as ITNs, where some household members might be preferentially protected [84–86], or in settings without mass distribution, where the poorest households are disproportionately less served. Additionally, LSM provides direct opportunities for community involvement by promoting job creation, citizen science, and local community participation and engagement. If residents can become engaged in source reduction efforts, LSM could further represent a more sustainable strategy for countries to reduce reliance on foreign funding, especially where the companies involved can provide high-quality products meeting essential regulatory approval requirements.

The approach to engaging households and communities matters, especially for interventions that require entry into people’s homes [87–89]. In a recent review that included social and behavioural considerations for LSM interventions, key themes included the importance of timely and regular engagement with communities and civil society, and community involvement in implementation. Cultivating trust, including through employment of community members, can increase acceptance and effectiveness of LSM interventions [90] (Hunter et al. in preparation). Hiring locally is key, but is not sufficient for some LSM interventions. While local employment can form a core aspect of broader community and stakeholder engagement, it will be context-dependent and distinct across settings.

An LSM programme might engage hundreds of people, both male and female, in various aspects of LSM implementation, thereby enhancing community acceptance and ownership. In an integrated management structure, teams could be employed to perform LSM during the non-IRS season, particularly in drier periods, and then transition to perform IRS or implement other interventions like spatial repellents or health education during the wetter seasons when the ratio of people to water bodies makes LSM less practical. This approach of re-skilling staff for various seasonal tasks was effectively demonstrated in the Magude Project in Maputo, Mozambique [91]. Ideally, this would create demand from the community to maintain the support of elected officials and potentially catalyse countries to put in place a centralized top-down structure to drive elimination. Endemic countries in the elimination phase should be supported in planning and implementing LSM at national and sub-national levels, with strong sub-national oversight and leadership where LSM could provide the final push towards elimination. For instance, the highly successful Dar es Salaam Urban Malaria Control program, a partnership between local government agencies and research and academic institutions, relied on hundreds of community-owned resource persons (CORPs) to support LSM [92]. This approach created a lasting workforce central to the continuation of the programme, and the CORPs themselves viewed their roles as professional, akin to employment rather than voluntary work [92]. The creators of this programme eventually recommended improving employment conditions, involving local health committees in staff recruitment and enhancing communication and community engagement skills to achieve effective community participation, particularly for accessing fenced and gated compounds in the city. They also suggested a simpler, more direct, community-based surveillance system managed by fewer, better-paid personnel to improve performance and data quality [92]. In low-income settings where percentage unemployment is commonly in the double digits and the population is rapidly growing, such LSM programmes can constitute significant local employment opportunities with multiple positive externalities.

To maximize impact, significant efforts should therefore be made to actively engage local populations, not only to obtain their consent and acceptance, but also to involve them in mapping, characterizing and prioritizing aquatic habitats [93], as well as in implementing, managing and evaluating selected LSM strategies. Additionally, while advancements in technology may improve habitat mapping and treatment, these technologies should complement, rather than replace, the involvement of the local workforce.

It is equally important to consider how communities depend on the same water bodies that harbour mosquitoes and to educate and involve them in decision-making regarding which LSM approaches to adopt. A recent study in Tanzania documented how community members use these habitats as a source of water for important daily activities such as cooking, drinking, washing utensils, washing clothes, bathing, crop farming, livestock rearing, brickmaking and fishing [94]. In this setting, the authors observed community readiness to implement LSM, favouring larviciding and habitat manipulation, which unlike habitat removal would preserve the water sources, though there were concerns about the safety of larvicides for animal and human health and their environmental impact. Other excellent examples of partnerships that exist between agriculture and malaria control efforts include the idea of farmer field schools to train staff to identify anopheline larvae alongside crop pest larvae to bolster surveillance [95]. These preferences might differ in other settings, but overall, the observations underscore the need for LSM strategies to consider both mosquito control and community needs, integrating educational efforts and culturally sensitive approaches.

To the degree possible, the engagement may also be extended to other relevant sectors to access experience or optimize resource use. For example, depending on the strategy adopted, LSM could preserve ecosystems by reducing the need for widespread use of insecticides, thereby reducing environmental impacts. Teams could also coordinate with agriculture to ensure more judicious use of pesticides, training subsistence farmers alongside communities on resistance [96]. The widespread use of chemical-based products for vector control began before the importance of environmentally protective practices was fully recognized. While *Bacillus*-based formulations for bio-larviciding are generally considered ecologically safe, concerns have been raised about their ecological impact [97]. LSM strategies like habitat mitigation and the use of larvivorous predators can provide sustainable mosquito control alternatives that produce less waste than commodity-based interventions (e.g. polyethylene or polypropylene waste from discarded nets). However, potential negative environmental impacts must be carefully assessed before introducing these approaches, given that some predatory fish, notably *Gambusia affinis*, can become invasive [98]. Additionally, cleaning up plastic waste, especially discarded tires that create breeding sites [99], and promoting responsible recycling are crucial for effective habitat removal [9, 100]. Such environmental sanitation has an added advantage, as it can improve control of *Aedes* vectors in places where risks exist but concerns focus on malaria transmission [101].

Lastly, education can be a powerful component of disease control operations and has played a pivotal role in the control of vector-borne diseases [102] including dengue [103], and has been widely adopted for malaria control by encouraging the use of ITNs [104–108] or through the use of outreach training and supportive supervision to improve the quality of service provision [109]. A recent review found that greater knowledge of larviciding increased support for its application [110, 111] (Hunter et al. in preparation). Expanding state education curricula to include vector control concepts, such as source reduction, will require tailored efforts that build on local knowledge and provide practical guidance to encourage sustained community actions for reducing larval habitat. While comprehensive knowledge can lay a valuable foundation for engagement and help to increase acceptance, it is not sufficient. It is also critical to understand and address context-specific barriers to participation and ensure that what is being asked of people is feasible in terms of the time, skills and resources required [112].

## Adopting technological advancements to improve feasibility and scalability of LSM

**Fig. 3:**
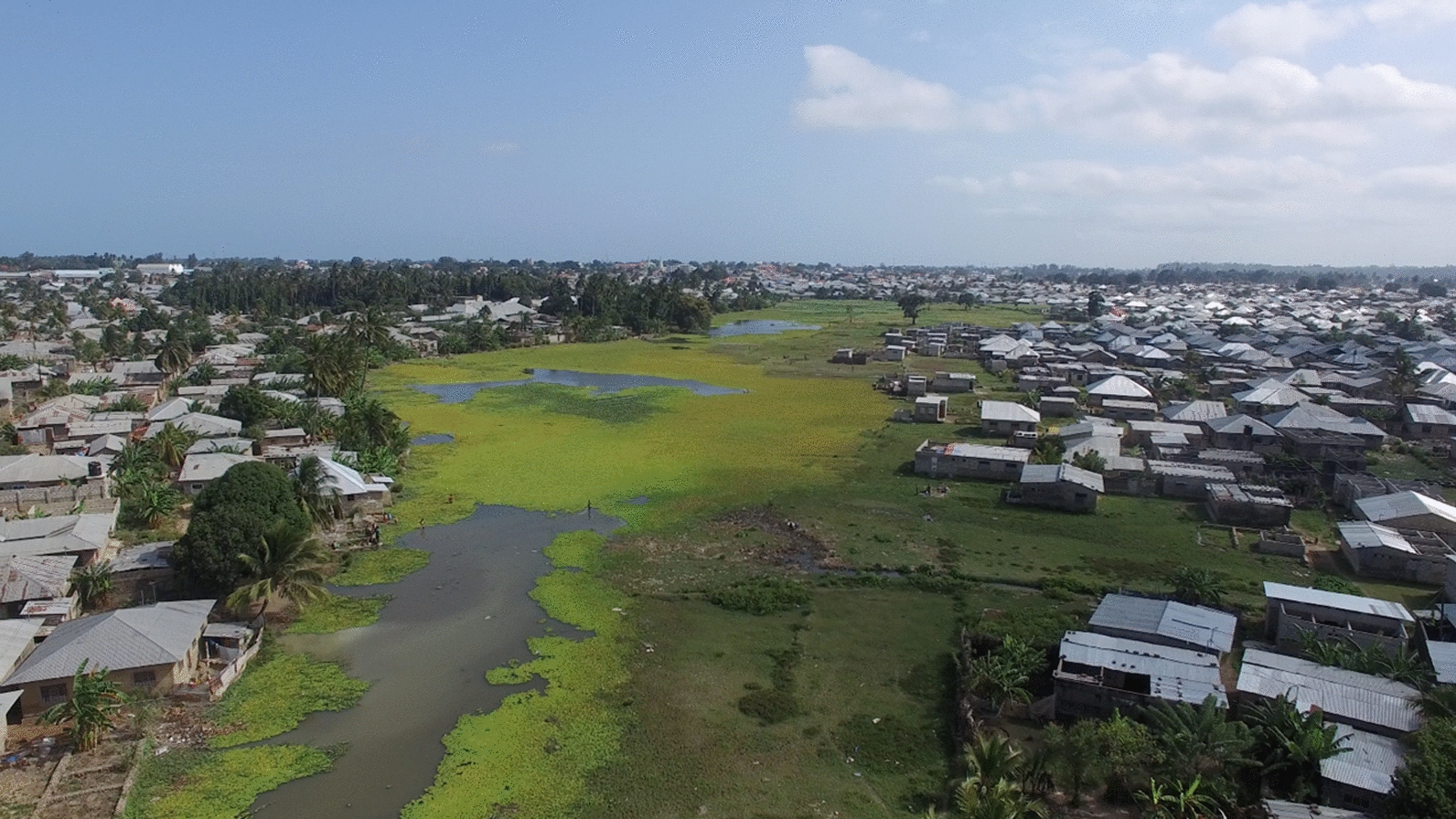
Aerial view of an extensive mosquito breeding site in Maboga, Zanzibar, captured from a low-cost drone

Most historical evidence supporting LSM is observational, similar to the extensive successes of IRS in the pre-ITNs era. These early successes in LSM were achieved before many of the technological advancements and commodities we have at our disposal today, such as drones, remote sensing, mobile applications and advanced geospatial analysis techniques (Fig. 3). There are further developments in equipment to deliver larvicides, some of which are liquid or granular mist blowers that can reach “cryptic” larval habitats (e.g. [113]). These developments are already showing the extended reach of LSM. For example, drones were used to map then deliver Bti larviciding to irrigated rice paddies in semi-urban Kigali, Rwanda, driving declines in larval and adult mosquito densities and community malaria burden [114]. These technological advancements could improve the planning of LSM and also ensure cost-efficient execution of the field operations [115], enabling success beyond what was already possible decades ago. However, the ultimate impact might vary by, among other factors, the nature of the technology, the eco-epidemiological settings and the technical know-how of the operators; thus, the cost-efficiency and impact of implementation urgently needs to be empirically tested [116].

The current WHO recommendations on LSM emphasize the importance of habitats being “few, fixed, and findable” [2]. We believe that the last item, “findable”, is the most critical factor, and that these guidelines could be simplified to habitats being simply findable, allowing them to be treated, modified or removed. Historical successes such as those in Brazil and Egypt [44] were made possible by substantial financial backing and a large local workforce. Today, advancements in artificial intelligence (AI), modelling, drones, smartphones and satellites present new opportunities to expand the number of habitats that are treatable. High-resolution aerial imagery from drones, multimodal data from satellite sensors and advanced image analysis techniques (including machine learning techniques), combined with ground-truthing data, can provide precise, accurate and timely information on the location of potential larval habitat. This facilitates the efficient scaling up of aquatic habitat mapping activities, which is a crucial precursor to deploying larviciding operatives.

Additionally, satellite imagery and hydrological terrain analysis can help target LSM activities [117]. Although these may not pinpoint individual larval habitat sites, they provide vital information to direct drone or field-based mapping efforts, potentially reducing financial costs for surveying and delivering LSM [115]. Hydrological mapping is particularly valuable for enhancing LSM strategies for malaria vector control in rural Africa. Key predictors of dry-season aquatic habitats, such as rivers and river channels, are essential for understanding the dispersal patterns and seeding of major vector species like *Anopheles funestus*, which dominate malaria transmission across most of the east and southern Africa region [118]. Field studies have shown that malaria transmission mediated by *An. funestus* can extend to the dry season, and therefore dry season control may be particularly effective in settings where this species dominates, especially if refugia are effectively targeted [119, 120]. In these settings, monitoring surface water dynamics over time helps classify water bodies as transient, ephemeral, seasonal or permanent, refining habitat targeting for vector control and maximizing resource allocation efficiency [117, 121]. In these contexts, using longer-lasting (and potentially more costly) larvicide formulations might be justified for permanent water bodies, whereas for transient and ephemeral ones, a less expensive, shorter-acting larvicide would suffice.

Apart from improving the capacity to find and target habitats through image analysis and other methods, these technologies could support researcher efforts to quantify and explicitly attribute impact to the LSM methods tested, potentially meeting WHO’s rigorous assessment criteria and allowing for broader recommendations. Mobile-based approaches can also be used for planning and monitoring the deployment, coverage and success of LSM activities [116], vastly improving the feasibility and effectiveness of LSM. Overall, technological advancements now make it easier to map, characterize and treat aquatic habitats. Emphasis should be placed on ensuring that any technology adoption is done to complement, rather than replace, the involvement of the local workforce.

Besides drones, mobile technologies, satellite imagery and the like, there have also been significant improvements in formulations of larvicides. To date, WHO has prequalified an extensive list of larvicide products [122], including some novel options with long-lasting activity. There is now a tablet form of a product isolated from the bacterium *Saccharopolyspora spinosa* that is reportedly active against container-breeding mosquitoes for up to 16 weeks depending on the dose [122, 123]. Another longer-lasting option for targeting container-breeding mosquitoes is 2%-pyriproxyfen Matrix Release (2MR) formulation that uses pyriproxyfen with active impacts reported for up to 6 months [122, 124]. The Bti strain AM65-52 WG also provides 8 weeks of residual effects when directly applied to containers [125, 126]. Adopting these formulations can greatly improve cost-effectiveness and logistical efficiency of larviciding operations, though if seasons are short then shorter-lasting and cheaper larvicides could be equivalently effective.

## Affordability, financing and sustainability of LSM

Current WHO guidelines, which broadly recommend larviciding only in urban and arid settings, are in part underpinned by the assumption that LSM strategies would not be cost-effective in other settings. However, larviciding has been shown to be broadly cost-effective across settings [127], and in some settings, the cost-effectiveness estimates were comparable to ITNs or IRS despite the need to continually treat larval habitat [128]. This approach has also been shown to significantly reduce malaria incidence, sometimes at levels equivalent to ITNs [50]. Although the cost of larvicides is not a major factor in economic evaluations compared to ITNs and IRS, addressing supply chain challenges and the small market for larvicides that limits both local manufacturing and scaled application (as market competition would drive down costs) is essential for improving the availability and affordability of quality larvicides. Alongside efforts to bolster local manufacturing, it is necessary to monitor and evaluate the quality of products. Addressing these issues through partnerships with in-country businesses and regulators and volume guarantees could improve supply and reduce costs. However, cost-effectiveness parameters are broadly context-specific and can be improved depending on specific strategies used. While further field data are needed, larviciding costs per person protected compare favourably with ITN campaigns [129], and may be even lower than IRS in semi-urban areas [128, 130]. Moreover, using longer-lasting formulations may further bring down the cost of operations by significantly reducing the treatment frequencies, larvicide quantities and workforce requirements.

Unfortunately, LSM has not received the kind of infrastructural investments that ITNs and IRS have enjoyed, which has hindered its broader adoption and has restricted opportunities for improving and evaluating cost-effectiveness. For both ITN and IRS, sustained investment has been made in developing efficient, adaptive and effective supply chains with well-defined distribution channels to the last mile. This, combined with straightforward outcome and impact evaluation metrics (such as coverage, number of structures sprayed, number of people protected and reduced disease burden), has resulted in more favourable perceptions among key stakeholders, including donors, policymakers and end-users. Private-sector companies do contribute to mosquito control, but quantifying the scale of impact from such efforts is a particular challenge [131, 132].

Besides larviciding, to our knowledge, there are no empirical epidemiological data or cost analysis for non-commodity LSM approaches, such as habitat mitigation, modification and the use of predatory species to reduce aquatic-stage mosquito numbers, which are still largely considered complex to implement and evaluate. Despite theoretical benefits shown in mathematical models ([27, 28], and see Fig. 2), a largely positive Cochrane review [39], and the frequent observational support for environmental sanitation (Fig. 1, details provided in Supplementary File 1), transparent and effective evaluation strategies are still lacking. Moreover, the effectiveness of source reduction varies by setting, potentially eliminating mosquitoes in low-burden areas but requiring more effort in high-burden areas (and this is the case for impacts from any vector control). Environmental sanitation through ecologically sensitive habitat modifications or removal requires significant upfront capital investment but can lead to permanent and sustainable benefits that last generations, making this approach more cost-effective over longer time periods. Yet it has to be recognized that any such action must be considerate of ecological harm [133]. Costs are geographically specific and access-dependent, explaining the challenges in universally recommending LSM without considering local socio-ecological factors and disease transmission determinants. We therefore recommend developing methods to assess longer-term cost-effectiveness of other LSM strategies like source reduction, which can permanently reduce breeding spaces and thus suppress mosquitoes without the need for regular replenishment of commodities, so that authorities can better understand whether the necessary upfront capital would maintain benefits and become continually more cost-effective as malaria cases would be averted over many years.

Lastly, to ensure sustainability, funding for LSM should be multifaceted, supported by both government agencies and private-sector contributions, with appropriate levels of integration into broader local programmes such as public health, environmental management and civil infrastructure. Examples of such shared funding models might include local governments financing the workforce, large equipment and products (e.g. larvicides) while private charities fund technological enhancements and auxiliary costs such as annual staff training and quality assurance. To begin with, national programmes should be encouraged to include requests for LSM funding within their grant applications to the Global Fund and other relevant donors, provided they can demonstrate impact and justification. Additionally, as part of the multi-sectoral approach, expertise should be drawn from relevant agencies including those responsible for environment, agriculture, sanitation and health. Bipartisan financial support and knowledge should be leveraged from each such stakeholder given the collective benefits for society. For example, the health ministry should focus on quality assurance and surveillance, while other sectors such as environment and civil works should conduct much of the LSM activities due to their relevant expertise. Lastly, in-country manufacturers of larvicides or LSM equipment, provided appropriate quality assurance and control is in place to ensure high quality, should be supported, for example by facilitating essential product evaluations and certifications and developing collaborative registration procedures between WHO prequalification and national regulatory authorities.

## Critical next steps in policy formulation for cross-sector integration, workforce development, and research and development

Where the national parent policy (and see [59]) already recommends LSM, in-country authorities should invest in diligent implementation and evaluation of the strategy and evidence to inform further expansions where appropriate. Broadly, there are already easily accessible workforces in most areas where mosquitoes are a health hazard. For instance, youth unemployment in sub-Saharan Africa is high, and employment of these young people to establish integrated habitat management could provide opportunities to absorb this large labour force productively and could alleviate both public health and economic challenges. Using local and remunerated labour for deployment of larvicides or other LSM activities not only fosters community engagement and ownership but also enhances the local knowledge base, as already demonstrated in Zanzibar [115]. Vector control technicians and environmental health officers can play a crucial role beyond larvicide deployment, requiring skills in sampling, ground-truthing remote sensing data and selecting LSM interventions and application methods [134]. It should be expected that these roles will necessitate significant training and appropriate compensation.

LSM should be recognized as a multi-sectoral strategy involving significant government participation, appropriate legal frameworks and collaboration across various sectors to leverage the expertise and resources necessary for its success. For example, expertise from civil and environmental engineering may be desirable for permanent habitat removal but is usually beyond the typical scope of health ministries. Funding may also often lie outside typical health ministry budgets, necessitating additional support from other sectors like environment, education, housing or civil works. Additionally, effective LSM impact demands stringent public health administration, cross-sector engagement, and large-scale mobilization, as demonstrated in countries like Palestine, Israel, the USA and Italy [47, 135]. Supportive legal framework and governance structures are therefore essential for enforcing mosquito control laws, integrating new technologies and fostering community participation.

Critically, there is need to develop a consensus on the evaluation process for impact of LSM. This might include how to monitor, evaluate and interpret the efforts required for successful LSM programmes—and should include both entomological and public health outcomes but also ecological outcomes such as suppression of insects that act as vectors or nuisance biters. Results of these evaluations should be made more widely available to inform expanded adoption of LSM. Box 2 outlines a priority research agenda that might be considered to support the scale-up of LSM in Africa. Priority research areas should include strategies for assessing the effectiveness of different forms of LSM, developing environmentally safe larvicide formulations with longer residual efficacy, integrating LSM with other vector control strategies and improving habitat management with advanced technologies like satellite imagery and drones. Additionally, investment is needed in cost-effective larvicidal application methods, optimizing community engagement and securing sustainable financing (Box 2).

**Box 2:** A priority research agenda to elevate LSM as a key strategy for vector control

1. Identify the applicability and efficacy of different LSM strategies—such as larviciding, habitat manipulation, habitat removal, and biological control—in African settings, particularly given the limited evidence beyond larviciding. Under what conditions are different approaches most effective and feasible?
2. Evidence challenges and opportunities that exist for integrating LSM with other vector control strategies, such as ITNs, IRS, and housing and urban development
3. Determine how technological advances, including satellite imagery and drones, citizen science, GIS and mobile technology—application equipment for area-wide treatment of cryptic larval habitats—can be leveraged to improve the identification and management of larval habitats across diverse geographical landscapes
4. Identify the most scalable and cost-effective methods for application of larvicides in rural and urban areas
5. Optimize community engagement within LSM initiatives to enhance local ownership and the overall effectiveness of malaria control efforts. Identify key behaviours to be carried out by individuals, households, and communities (and factors that influence those behaviors)
6. Develop strategies to ensure sustainable financing and support for LSM from local governments and private sectors
7. Quantify the impact of LSM on reducing vector populations in rural African settings and consequences for epidemiological outcomes
8. Assess environmental hazards of LSM, particularly regarding chemical use, and mitigate to minimize negative impact
9. Integrate datasets (e.g. hydrological, topographic and entomological data) to improve the precision of habitat identification and management, and identify necessary additional data sources
10. Establish best practices for tailored LSM treatment schedules that align with local ecological conditions and maximize effectiveness
11. Explore effective implementation of LSM for sustained impact in the context of climate change, protection of biodiversity, new infrastructure development and increasingly unpredictable weather patterns

## Conclusions

The stagnation of progress in the control of malaria and other vector-borne diseases necessitates the re-evaluation and scaling up of LSM as a key component of integrated vector control strategies, particularly in Africa. LSM has proven historical success, and recent technological advancements improve its feasibility in future efforts. An update of LSM guidelines is needed to capture the existing high-quality operational evidence and to recognize the advantages provided through advanced technologies and our growing understanding of disease vector bionomics. Key stakeholders should prioritize the development of a consensus framework for LSM evaluation, and engage across multiple sectors and ministerial departments. In doing so, communities can benefit fully from the comprehensive expertise and capacity of regions. Enhanced community engagement through local workforces can deliver equitable, effective and sustained implementation of LSM with potentially transformative results for vector-borne disease control and society.

## Supplementary Information

Supplementary Material 1.

Supplementary Material 2.

## Data Availability

Data are provided within the manuscript or supplementary information files.
